# Allergic multimorbidity is associated with self‐reported anaphylaxis in adults—A cross‐sectional questionnaire study

**DOI:** 10.1002/clt2.12184

**Published:** 2022-07-21

**Authors:** Tuuli Thomander, Sanna Toppila‐Salmi, Johanna Salimäki, Juha Jantunen, Heini Huhtala, Paula Pallasaho, Paula Kauppi

**Affiliations:** ^1^ Doctoral Programme in Clinical Research University of Helsinki Helsinki Finland; ^2^ Department of Allergology Skin and Allergy Hospital Helsinki University Hospital and University of Helsinki Helsinki Finland; ^3^ Association of Finnish Pharmacies Helsinki Finland; ^4^ South Karelia Allergy and Environment Institute Imatra Finland; ^5^ Faculty of Social Sciences Tampere University Tampere Finland; ^6^ The Heart and Lung Center Helsinki University Hospital University of Helsinki Helsinki Finland

**Keywords:** allergic rhinitis, anaphylaxis, asthma, atopic dermatitis, food allergy

## Abstract

**Background:**

Anaphylaxis has increased over the last two decades in Europe, reaching an estimated prevalence of 0.3% and an incidence of 1.5–7.9 per 100,000 person‐years. Allergic multimorbidity is associated with asthma severity, yet its role in anaphylaxis is not fully understood. Our aim was to study association between allergic multimorbidity and anaphylaxis in adults.

**Methods:**

We used population‐based data from the Finnish Allergy Barometer Study (*n* = 2070, age range: 5–75). Food allergy (FA), atopic dermatitis (AD), allergic rhinitis (AR) and allergic conjunctivitis (AC), were defined from a self‐completed questionnaire. A logistic regression adjusted on potential confounders (sex, age, smoking status) was applied to estimate the anaphylaxis risk associated with allergic multimorbidity.

**Results:**

1319 adults with at least one allergic disease (FA, AD, AR, AC) with/without asthma (AS) were included. Of these, 164 had self‐reported anaphylaxis [mean (SD, min‐max) 54 (14, 22–75) years, 17% men]. AS, FA, AR, AC, or AD were reported by 86.0%, 62.2%, 82.3%, 43.3%, and 53.7% of subjects with anaphylaxis and respectively by 67.8%, 29.5%, 86.2%, 29.4%, and 34.4% of subjects without anaphylaxis. Compared with subjects exhibiting only one allergic disease, the risk of anaphylaxis increased with the number of allergic diseases; adjusted odds ratios (OR) [CI95%] for two, three, four and five coinciding allergic diseases were 1.80 [0.79–4.12], 3.35 [1.47–7.66], 7.50 [3.25–17.32], and 13.5 [5.12–33.09], respectively. The highest risk of anaphylaxis (6.47 [4.33–9.92]) was associated with FA + AS or their various variations with AR/AC/AD embodied, when compared with AR, AC, and AS separately or their combinations.

**Conclusions:**

Anaphylaxis was positively associated with the number of allergic diseases a subject exhibited and with subgroups including FA and/or AS. The results can be applied when estimating the risk of anaphylaxis for individual patients.

## 1 BACKGROUND

Anaphylaxis is a severe, potentially life‐threatening systemic hypersensitivity reaction,^1^ including rapid onset with life‐threatening airway, breathing, or circulatory problems. It is usually, although not always, associated with skin and mucosal changes. The prevalence of anaphylaxis has increased over the last two decades and is currently approximately 0.3% in Europe. The incidence is 1.5–7.9 per 100,000 person‐years.^2^ From 2008 to 2018, an annual increase of 5.9% in hospital admissions was reported in the United Kingdom due to anaphylaxis among patients 15–65 years of age.^3^ Increasing trends of hospitalizations and incidence of anaphylaxis have been reported internationally.^4^, ^5^ The case fatality rate is low, but anaphylaxis deaths are still reported.^2^, ^4^ The incidence of anaphylaxis deaths is 0.59 per million person‐years in Finland and has slightly increased from 1996 to 2013.^6^ However, the incidence of fatal anaphylaxis remained the same in the United Kingdom between 1992 and 2012.^7^ The burden of anaphylaxis has remarkable effects on health and costs.^8^

Food is the most common cause for anaphylaxis in both children and adults but is more frequent in the pediatric than the adult population. Furthermore, no difference was reported in the frequency of insects as triggers or idiopathic etiology between children and adults. However, drug‐induced anaphylaxis was more frequent in the adult population.^9^ Risk factors for severe anaphylaxis in children were recently shown to embody a history of asthma (AS), which is in controversy with previous suggestions.^10^ Blöndal et al. (2021) showed that multimorbidity including AS, rhinitis, and eczema in adults increased the risk of severe allergic reactions for anaphylaxis to food.^11^

In a US study, food was a trigger for anaphylaxis in 48%, drugs in 30%, insects in 3% and the trigger remained unknown in 19% of the cases.^9^ The severity of anaphylactic symptoms and multiple epinephrine doses were predictive of hospitalization in adults whereas chronic pulmonary disease was not.^9^ In an earlier study, medication‐related anaphylaxis (odds ratio (OR) 1.50), age of at least 65 years (OR 3.15), cardiac disease (OR 1.56) or lung disease (OR 1.23) were predictive of severe anaphylaxis and hospitalization.^12^ Further, recurrent anaphylaxis is a rare phenomenon, and only 3.0% of those visiting emergency department do so because of anaphylaxis.^13^ Food allergy (FA), AS (OR 1.30) and a history of intensive care unit admission were found to be risk factors for a new emergency department visit caused by anaphylaxis.^13^

Key triggers for anaphylaxis comprise food, drugs, and stinging insects.^2^, ^9^ However, their relative importance varies with age and geography. Allergic diseases and AS are the greatest risk factors in children, whereas drugs and stings are more frequent risk factors among adults than in children. Data are lacking concerning the magnitude of each risk factor for anaphylaxis.^1^

Several cohort studies have showed that allergic diseases are associated with an increased risk of AS in children^14^, ^15^ and in adults.^11^ Allergic multimorbidity has been shown to be associated with disease severity in elderly French women,^16^ and the number of allergens to which a child is sensitized is related to the severity of IgE‐mediated symptoms in children.^17^ AS, rhinitis and eczema together are a higher risk for seasonal allergy than AS only. Further, food allergies were more common in those with all three allergic diseases compared with those with AS only.^11^

The early detection of anaphylaxis risk factors is important for improving patient counselling and reducing anaphylaxis morbidity and mortality. As knowledge remains limited concerning the role of allergic multimorbidity in anaphylaxis in adults, our aim was to assess the association between allergic multimorbidity and anaphylaxis in a population‐based study. We hypothesized that the risk of anaphylaxis increases with the number of allergic multimorbidities (AS, FA, atopic dermatitis (AD), allergic rhinitis (AR), allergic conjunctivitis (AC)). Our secondary hypothesis was that anaphylaxis risk differs between various combinations of allergic multimorbidity.

## 2 METHODS

### 2.1 Study design

This is a cross‐sectional Finnish Allergy Barometer Study. We used a questionnaire of allergy and/or AS, with responses gathered in 2010 and 2016.

### 2.2 Setting

Customers purchasing allergy or AS drugs in Finland. A total of 100 pharmacies around Finland were involved.

### 2.3 Study population

The Finnish Allergy Barometer Study was conducted as a structured questionnaire. Inclusion criteria encompassed customers of 5–75 years of age purchasing allergy or AS drugs prescribed by a physician. The study was conducted during a one‐week period in September 2010 or 2016 in the participating pharmacies.^18^, ^19^, ^20^ Participating pharmacies were not obligated to participate during both years, and we assumed that subjects did not respond twice to the questionnaire. The study populations constituted altogether 2070 respondents in 2010 (*n* = 1114) and 2016 (*n* = 956). If needed, the pharmacy personnel assisted the respondents with the practicalities of completing the questionnaire. The questionnaire survey was conducted following international guidelines for epidemiological studies.^21^ The study was approved by the Ethical Committee of Helsinki University Hospital and Uusimaa Hospital District (153/13/03/01/2010). The data were stored and analysed anonymously.

**FIGURE 1:**
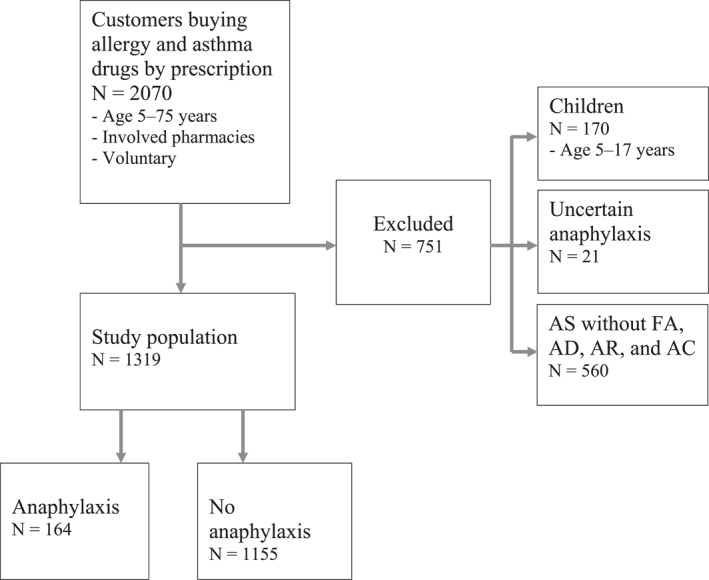
A flow chart of the study population selection. FA = food allergy, AD = atopic dermatitis, AR = allergic rhinitis, AC = allergic conjunctivitis. Uncertain anaphylaxis = study individual with anaphylaxis not meeting inclusion criteria

We examined risk factors for anaphylaxis in an adult population exhibiting at least one allergic disease (AR/AC/AD/FA). Exclusion criteria were age <18 years, AS with no FA, AD, AR, or AC, and additionally anaphylaxis with no FA, AD, AR, or AC. We wanted to focus on allergic asthma. We supposed that patient with allergic asthma would have also reported FA, AD, AR or AC. The total number of subjects was 1319 respondents, 164 of whom suffered from anaphylaxis (Figure 1).

### 2.4 Outcome and variables

Anaphylaxis was the outcome. Variables of interest were allergic multimorbidity (presence of AS/FA/AD/AR/AC). Other variables (putative confounding factors) were smoking status, gender, and age.

The following question was used to determine a diagnosis of anaphylaxis, AS, FA, AD, AR, and AC: “Do you have, or have you had **physician**‐diagnosed: (1) asthma (2) allergic rhinitis (3) allergic conjunctivitis (4) atopic dermatitis (5) food allergy (6) anaphylaxis (a severe allergic reaction)”. The respondents were required to circle the correct option. No instructions were given to circle the correct number of options pertaining to each subject. We assumed the respondent would circle all suitable options.

We established allergy multimorbidity subgroups based on reported combinations of allergic diseases (AS, FA, AD, AR, AC) by each study individual and used them alone or in different combinations in models investigating their association with anaphylaxis.

We formed four sections of allergic multimorbidity combinations:

Section 1: AR, AC, AS or their combinations.

Section 2: AD and its different combinations with AR/AC/AS except for those included in Sections 3 and 4.

Section 3: FA or different combinations with AR/AC/AD, except for those included in Section 4.

Section 4: FA + AS and their different combinations with AR/AC/AD.

Smoking (ever, never) was determined by the question: “Do you smoke?”. Response options were: 1) I have never smoked 2) Yes, occasionally 3) Yes, regularly 4) I have quit smoking.

Missing values were recoded as ‘no’ for all variables.

### 2.5 Statistical methods

Comparisons of the demographic data or allergic multimorbidity prevalence between anaphylaxis and non‐anaphylaxis groups were performed with the Mann‐Whitney *U* test (continuous variables) and Chi‐square test (categorical variables). For multivariate comparisons, binary logistic regression models were used and were reported as ORs with 95% confidence intervals (CIs). The association between allergic diseases and anaphylaxis was examined considering three consecutive allergic multimorbidity variables (i.) type of allergic disease, to test whether each allergic multimorbidity confers an anaphylaxis risk of similar magnitude; (ii) the number of allergic multimorbidities to address a possible dose–response relationship (iii.) subgroup of allergic disease, to test whether subgroup affects the anaphylaxis risk of similar magnitude. All models were adjusted by age, gender, and smoking status. Statistical analyses were conducted with SPSS Statistics 27 (IBM, 2020) software. All tests were two‐sided and a *p*‐value of 0.05 or less was considered statistically significant.

## 3 RESULTS

The study population embodied 164 anaphylaxis subjects (12.4%) and 1155 study individuals without anaphylaxis. AS, FA, AR, AC or AD were reported by 86.0%, 62.2%, 82.3%, 43.3%, 53.7% of subjects with anaphylaxis and respectively by 67.8%, 29.5%, 86.2%, 29.4%, 34.4% of subjects without anaphylaxis (Table 1). Thus, the proportion of AS, FA, AC, and AD were significantly higher in the anaphylaxis group than in the non‐anaphylaxis group, whereas not difference was found with the proportion of AR (Table 1). Furthermore, the subjects with anaphylaxis reported higher age [mean (SD, min–max) 54 (14, 22–75) years versus 49 (16, 18–75 years, *p* < 0.001) and more probably had a history of smoking at some point during their lives (yes 46% vs. 38%, *p* < 0.05) (Table 1), whereas not gender differences were observed between the groups. Of the patients with anaphylaxis 44% reported emergency room visits due to allergies or asthma in the past 12 months (Table S1).

We observed a dose–response relationship between the number of allergic diseases and anaphylaxis: the adjusted OR [CI95%] for two, three, four and five allergic diseases were 1.80 [0.79–4.12], 3.35 [1.47–7.66], 7.50 [3.25–17.32], and 13.5 [5.12–33.09], compared with one allergic disease. If food allergy was left out and the dose‐response relationship between the number of allergic diseases and anaphylaxis was calculated the adjusted OR [CI95%] for two, three and four allergic diseases were 1.17 [0.68–2.02], 2.13 [1.2–3.7], 5.42 [2.95–9.95] (Table 2).

**FIGURE 2:**
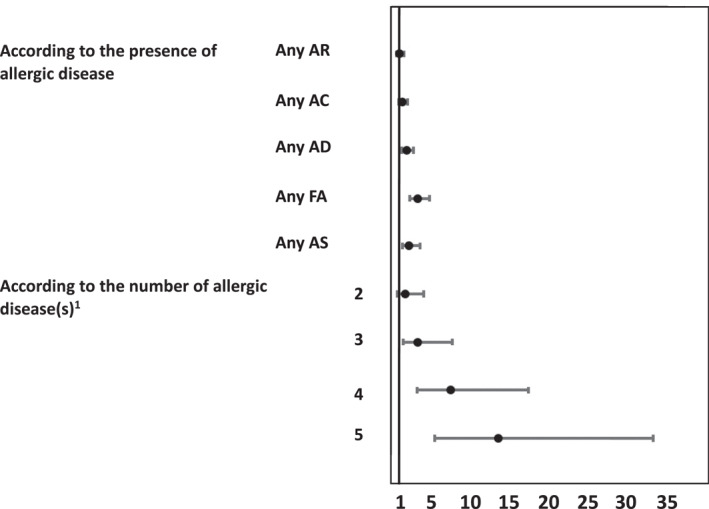
Adjusted odds ratio (OR) and 95%CI of self‐reported anaphylaxis ever are presented for the following allergic diseases (allergic rhinitis (AR), allergic conjunctivitis (AC), atopic dermatitis (AD), food allergy (FA), and asthma (AS)) and for the number of allergic diseases. Models were adjusted to gender, age, and smoking. All subjects had at least one allergic disease. The total number of subjects is 1319 and those with anaphylaxis occurrence is 164. The number of times an allergic disease occurred among subjects with self‐reported anaphylaxis: AR = 135, AC = 71, AD = 88, FA = 102, AS = 141. ^1^One allergic disease was the reference value with OR = 1.

In the adjusted models, higher age and the presence of the following allergic diseases were statistically significantly associated with a higher risk of anaphylaxis: AS (2.91 [1.84–4.60]), FA (3.93 [2.80–5.52]) and AD (2.21 [1.59–3.08]) (Figure 2, Table 3). Sex, smoking status, or the presence of AR or AC were not statistically significantly associated with anaphylaxis (Table 3). Each combination of allergic disease(s) was entered into a univariate model to evaluate its association with anaphylaxis. The following combinations were positively associated with anaphylaxis: AS + AR + AC + AD (2.2 [1.11–4.48]), AS + AF (2.11 [1.06–4.22]), AS + AR + FA (1.87 [1.06–3.33]), AS + AR + AD + FA (3.35 [1.88–5.99]), AS + AR + AC + AD + AF (4.56 [2.80–7.42]), when compared with a group that did not exhibit these combination (Table S2). The following disorders were negatively associated with anaphylaxis: AR (0.18 [0.07–0.50]), AS + AR (0.37 [0.23–0.61]) (Table S2).

We finally observed the different combinations (“sections”) of allergic diseases in the adjusted models. Section 4 (FA + AS or their variations with AR/AC/AD) embodies the highest risk of anaphylaxis (6.47 [4.33–9.92]) compared with Section 1 (AR, AC, AS, or their combinations) (Table 4). Section 3 (FA or its variations with AR/AC/AD) showed the second highest risk of anaphylaxis (2.65 [1.28–5.50]) and Section 2 (AD or its variations with AR/AC/AS) had the third highest risk (2.22 [1.31–3.76]) compared with individuals in Section 1 (Table 4).

## 4 DISCUSSION

We demonstrated a strong association between allergic multimorbidity and self‐reported anaphylaxis in adulthood. Our major finding was that anaphylaxis was positively associated with the number of allergic diseases, and with the subgroups of allergic diseases consisting of FA and/or AS. This is in line with a previous cross‐sectional survey in Sweden embodying 437 adults with AS, which reported that concomitant AS, rhinitis, and eczema increased the risk of severe allergic reactions to food.^11^ Adult patients with AS, rhinitis and eczema experienced more severe FA reactions (fainting, respiratory distress) (28% vs. 10%) than subjects with AS only. Furthermore, subjects with AS, rhinitis, and eczema were more frequently sensitized to seasonal allergens (67% vs. 32%), food allergens (54% vs. 18%), and had a higher degree of sensitization than subjects with AS only.^11^ Counselling patients of allergic multimorbidity as an anaphylaxis risk, and their more intensive follow‐up could decrease suffering due to anaphylaxis.

Risk factors for anaphylaxis may include FA (especially peanuts and tree nuts, and cow's milk allergy in children are risk factors for severe anaphylaxis)^22^ and the FA + AS combination,^23^ uncontrolled AS, AR, AD, AC, host‐related factors such as alcohol, risk‐taking, medication, and exercise,^22^ yet these are not fully understood. Prior studies on allergic multimorbidity and anaphylaxis in adults are lacking. The association between allergic multimorbidity and food‐related symptoms has been studied in adolescents with food‐induced anaphylaxis in the population‐based birth cohort (*N* = 3153, follow‐up period 0–16 years of age). The study found that AS, rhinitis, eczema, and sensitization to food and airborne allergens at age 16 were significantly associated with food‐related symptoms, with a stronger association among adolescents with anaphylaxis than those without.^24^

We found that AS, FA, AR, AC or AD were reported by 86%, 62%, 82%, 43%, or 54% of subjects with anaphylaxis and respectively by 68%, 30%, 86%, 29%, or 34% of subjects without anaphylaxis. In a Polish retrospective study of 10,738 patients (age range 0–76 years) with suspicion of any allergy or non‐allergic hypersensitivity 24% of patients with moderate or severe anaphylaxis exhibited AR as a comorbidity, 23% exhibited AS, 1% exhibited atopy, and only 2% exhibited AD.^25^ Thus, the proportion of AD was higher in our study than in the previous literature. This could be explained by the different study populations.

We showed that three or more allergic diseases increased the risk of anaphylaxis. The results remained similar also when food allergy was left out from the analyses. This dose–response effect of allergic multimorbidity has previously been detected in a Finnish cross‐sectional population‐based case‐control study with 1118 cases of AS (age range 30–93 years) and 1772 matched controls exhibiting adult‐onset as the endpoint.^26^ The study showed that adult‐onset AS was positively associated with the number of allergic diseases, and the association decreased with age.^26^ The association between AS and allergic multimorbidity has been recognized in children in the MeDALL study (an integrated study of 14 European birth cohorts with 44,010 participants), where the coexistence of eczema, rhinitis, and AS were more common than expected by chance alone.^14^ The Polish study showed that adult patients visiting hospital due to anaphylaxis statistically have one other disease.^25^ This may be due to a different selection of comorbidities and cofactors, and hence e. g. cardiovascular diseases, thyroid disease, and diabetes were included with remarkable percentages. Additionally, rhinitis was not specified as AR, and AD appeared in only 1.8% of cases while for example, cardiovascular diseases appeared in 31.4%.^25^

In our study, higher age and AS, FA, AD, AC, and a history of smoking were associated with the anaphylaxis group when compared with the non‐anaphylaxis group, whereas AR and female gender were not associated with anaphylaxis. A systematic review of 59 articles investigating the global epidemiology of anaphylaxis in the general paediatric population demonstrated that gender is associated with anaphylaxis in children. Males have a higher incidence rate of anaphylaxis in under 10‐year‐olds, while females have a higher incidence rate than males from 10 years of age onwards.^5^ However, gender was not a risk factor for anaphylaxis in our study examining the adult population. Our observation of age could be due to our questionnaire setup, which enquired whether respondents had ever exhibited anaphylaxis and allergic multimorbidity. The cumulative risk of anaphylaxis therefore increases with increasing age.

According to our results, the more allergic diseases reported the higher the risk for anaphylaxis. Additionally, we recognized more multimorbid‐polysensitized phenotypes. Of these, the FA + AS combination and its variations with AD, AR, and AC embodies the most severe phenotype. This agrees with previous findings of more severe symptoms in multimorbid‐polysensitized subjects in a study of 1199 adult subjects from a French 20‐year follow‐up study combining a case‐control and a family‐based study of AS cases. The study described more severe that nasal symptoms and more common eczema in patients with both AS and AR than in patients with only AS or AR.^27^ A cross‐sectional study of Korean school children (*N* = 3368, age range 6–7 years) reported a poor association with aeroallergen polysensitization and allergic multimorbidity. However, an association was found between a greater number of sensitized allergen classes and an increased risk for wheezing; adjusted OR 2.2 for one allergen, 2.8 for two allergens, 5.9 for three allergens, and 9.4 for at least four allergens.^17^

AS, FA, AC, and AD were significantly higher in the anaphylaxis group than in the non‐anaphylaxis group, whereas no difference was found with the proportion of AR. The French study of allergic sensitization patterns associated with AS, rhinitis, and their multimorbidity in adults showed polysensitization to be highest among subjects with AS + AR when compared with AS or rhinitis alone.^27^ In addition, nasal symptoms were more severe in participants with AS + AR than AS or AR separately or AS combined with non‐allergic rhinitis, which was in line with the findings in the MeDALL study for adults.^14^, ^27^ The authors noted that allergic sensitization is not a dichotomic variable and the multimorbid‐polysensitized phenotype could constitute a specific phenotype. Demands were also expressed for further studies of classifying allergic phenotypes.^27^

According to the German follow‐up study, single resting lung function values do not seem to discriminate well from early school age to young adulthood in study individuals with allergic multimorbidity and single allergies, while bronchial hyperresponsiveness was clearly more frequent and severe with allergic multimorbidity (AS + AR) subjects than those with AS or AR only.^28^ This supports our result that FA + AS is the most high‐risk allergic multimorbidity combination for anaphylaxis. Allergic multimorbidity studies including FA are lacking. However, a Polish multicentre cross‐sectional study on children and adolescents (age range 6–18 years) reported that FA is more frequent in children with allergy multimorbidity (other variables: AS, AD, AR) than those with one allergic disease.^29^ In addition, multimorbidity in early life and food‐related symptoms in later life have been indicated to have a strong association. Adolescents with anaphylaxis have more frequently exhibited polysensitization, including for example, several foods and nut storage proteins when compared with adolescents without anaphylaxis.^24^

Previous research highlights the small number of allergy comorbidity studies in adult populations,^15^ and there has been demand for further epidemiological studies of allergic phenotypes.^27^ Moreover, information concerning allergic comorbidity in adults and the magnitude of adult anaphylaxis risk factors have been lacking.^1^, ^15^ Furthermore, previous allergic multimorbidity studies mainly cover the variables AS, AD, and AR, and infrequently also AC. Information of FA as part of allergic multimorbidity is limited, yet FA is a remarkable risk factor for anaphylaxis.

The strength of our study is that we used a wide national data population of adult patients with anaphylaxis and allergic diseases, and that the data cover all five allergy phenotypes: AS, AR, AC, AD, and FA. It is possible, yet unlikely that an individual would have participated twice in this questionnaire study. The data was collected anonymously and therefore we are not able to track this. The data was collected in September for practical reasons. If data would have been collected e. g. in May, the number of study individuals with allergic rhinitis would potentially have been overrepresented. The number of patients with anaphylaxis is higher than usual. However, the study population constitutes only patients purchasing prescribed allergy or asthma medication. In general, patients with anaphylaxis often have allergic diseases. When compared with population‐based studies this is reasonable.^2^

The weakness of our study constitutes the self‐reported data. For example, objective lung function and allergy testing are therefore missing. However, physician‐made diagnoses have been shown to be reliable in Finland in a general adult population‐based study (*N* = 292).^30^ Food is one of the triggers of the anaphylaxis, but food allergy is also allergic disease and therefore we wanted to include it in this allergic multimorbidity study. We acknowledge that self‐reported FA includes cases reporting oral allergy syndrome and not a severe true FA. We also supposed that subjects without reported AR, AC, AD, and FA had non‐allergic AS and were therefore excluded. Missing cases were recorded as ‘no’ and might give response bias. The cross‐sectional design does not allow asserting the causal direction of associations between allergic multimorbidity and anaphylaxis. However, this pilot study may provide interesting information worth further longitudinal studies.

## 5 CONCLUSIONS

Self‐reported anaphylaxis in adults was positively associated with the number of allergic diseases, and with the subgroups of allergic diseases consisting of FA and/or AS. The results of our study can be applied when estimating the anaphylaxis risk for individual patients in the clinic and to better counsel at‐risk patients. Further longitudinal studies are still needed.

## AUTHOR CONTRIBUTIONS

**Tuuli Thomander**: Formal analysis (equal); Methodology (equal); Visualization (equal); Writing – original draft (equal); Writing – review & editing (equal). **Sanna Toppila‐Salmi**: Conceptualization (equal); Methodology (equal); Visualization (equal); Writing – review & editing (equal). **Johanna Salimaki**: Conceptualization (equal); Investigation (equal); Methodology (equal); Writing – review & editing (equal). **Juha Jantunen**: Conceptualization (equal); Investigation (equal); Methodology (equal); Writing – review & editing (equal). **Heini Huhtala**: Writing – review & editing (equal). **Paula Pallasaho**: Writing – review & editing (equal). **Paula Kauppi**: Conceptualization (equal); Investigation (equal); Methodology (equal); Writing – review & editing (equal).

## CONFLICT OF INTEREST

The authors declare that they have no competing interests.

## CONSENT FOR PUBLICATION

Not applicable.

## Supporting information

Supporting Information S1

## APPENDIX A

**TABLE 1:** Study characteristics

	No anaphylaxis	Anaphylaxis	*p* value
	(*n* = 1155) *n* (%)	(*n* = 164) *n* (%)	
Any^a^ AR			
No	159 (13.8)	29 (17.7)	0.189
Yes	996 (86.2)	135 (82.3)	
Any^a^ AC			
No	816 (70.6)	93 (56.7)	**<0.001**
Yes	339 (29.4)	71 (43.3)	
Any^a^ AD			
No	758 (65.6)	76 (46.3)	**<0.001**
Yes	397 (34.4)	88 (53.7)	
Any^a^ FA			
No	814 (70.5)	62 (37.8)	**<0.001**
Yes	341 (29.5)	102 (62.2)	
Any^a^ AS			
No	372 (32.2)	23 (14.0)	**<0.001**
Yes	783 (67.8)	141 (86.0)	
Age			
Mean	48.6	54.4	
Median	49.0	58.0	
SD	15.6	13.8	**<0.001**
Min	18	22	
Max	75	75	
Female			
No	234 (20.3)	27 (16.5)	0.295
Yes	921 (79.7)	137 (83.5)	
Smoking history			
Never	714 (61.8)	88 (53.7)	**0.049**
Yes, at some point	441 (38.2)	76 (46.3)	

*Note*: Bold values indicate *p* value of 0.05 or less was considered statistically significant.

Abbreviations: AC, allergic conjunctivitis; AD, atopic dermatitis; AR, allergic rhinitis; AS, asthma; FA, food allergy; SD, standard deviation.

^a^ The specific condition appears alone or with any combination of other allergic diseases (allergic rhinitis, allergic conjunctivitis, atopic dermatitis, food allergy and asthma, except from the one observed).

**TABLE 2:** The anaphylaxis risk and the number of allergic diseases

	Univariate		Multivariable		Univariate without FA		Multivariable without FA	
Risk factor	OR (CI 95%)	*p* value	OR (CI 95%)	*p* value	OR (CI 95%)	*p* value	OR (CI 95%)	*p* value
Number of allergic diseases								
One	1.00		1.00		1.00		1.00	
Two	2.06 (0.91–4.66)	0.084	1.81 (0.79–4.12)	0.159	1.24 (0.72–2.13)	0.438	1.17 (0.68–2.02)	0.570
Three	3.57 (1.57–8.11)	**0.002**	3.35 (1.47–7.66)	**0.004**	2.20 (1.26–3.85)	**0.006**	2.13 (1.21–3.75)	**0.009**
Four	7.94 (3.46–18.22)	**<0.001**	7.50 (3.25–17.32)	**<0.001**	5.62 (3.09–10.23)	**<0.001**	5.42 (2.95–9.95)	**<0.001**
Five	13.70 (5.67–33.10)	**<0.001**	13.51 (5.52–33.09)	**<0.001**				
Age	1.03 (1.01–1.04)	**<0.001**	1.03 (1.02–1.04)	**<0.001**	1.03 (1.01–1.04)	**<0.001**	1.03 (1.01–1.04)	**<0.001**
Female	1.29 (0.83–2.00)	0.255	1.10 (0.69–1.75)	0.689	1.29 (0.83–2.00)	0.255	1.15 (0.73–1.83)	0.545
A history of smoking	1.40 (1.01–1.94)	**0.046**	1.36 (0.96–1.94)	0.086	1.40 (1.01–1.94)	**0.046**	1.37 (0.97–1.93)	0.078

*Notes*: Diseases: allergic rhinitis, allergic conjunctivitis, atopic dermatitis, food allergy, asthma. Bold values indicate *p* value of 0.05 or less was considered statistically significant.

**TABLE 3:** The anaphylaxis risk of each risk factor

	Univariate		Multivariable	
Risk factor	OR (CI 95%)	*p* value	OR (CI 95%)	*p* value
Any^a^ AR	0.74 (0.48–1.15)	0.181	1.02 (0.64–1.64)	0.920
Any^a^ AC	1.84 (1.32–2.57)	**<0.001**	1.43 (0.98–2.08)	0.063
Any^a^ AD	2.21 (1.59–3.08)	**<0.001**	1.96 (1.36–2.81)	**<0.001**
Any^a^ FA	3.93 (2.80–5.52)	**<0.001**	3.40 (2.37–4.88)	**<0.001**
Any^a^ AS	2.91 (1.84–4.60)	**<0.001**	2.26 (1.40–3.65)	**0.001**
Age	1.03 (1.01–1.04)	**<0.001**	1.03 (1.02–1.04)	**<0.001**
Female	1.29 (0.83–2.00)	0.255	1.12 (0.70–1.80)	0.643
A history of smoking	1.40 (1.01–1.94)	**0.046**	1.34 (0.94–1.91)	0.110

*Note*: Bold values indicate *p* value of 0.05 or less was considered statistically significant.

Abbreviations: AC, allergic conjunctivitis; AD, atopic dermatitis; AR, allergic rhinitis; AS, asthma; FA, food allergy.

^a^ The specific condition appears alone or with any combination of other allergic diseases (allergic rhinitis, allergic conjunctivitis, atopic dermatitis, food allergy and asthma, except from the one observed).

**TABLE 4:** The anaphylaxis risk for the allergic multimorbidity subgroup sections exhibited in the study population

	No anaphylaxis	Anaphylaxis	Univariate		Multivariable	
	*n* (%)	*n* (%)	OR (CI 95%)	*p* value	OR (CI 95%)	*p* value
Total	1155 (100.0)	164 (100.0)				
Section 1: AR, AC, AS, or their combinations						
No	575 (49.8)	130 (79.3)	1.00		1.00	
Yes	580 (50.2)	34 (20.7)				
Section 2: AD and different combinations with AR/AC/AS						
No	921 (79.7)	136 (82.9)	0.81 (0.53–1.25)	0.339	2.22 (1.31–3.76)	**0.003**
Yes	234 (20.3)	28 (17.1)				
Section 3: FA and different combinations with AR/AC/AD						
No	1063 (92.0)	153 (93.3)	0.83 (0.44–1.59)	0.575	2.65 (1.28–5.50)	**0.009**
Yes	92 (8.0)	11 (6.7)				
Section 4: FA + AS and their different combinations with AR/AC/AD						
No	903 (78.2)	72 (43.9)	4.54 (3.23–6.36)	**<0.001**	6.47 (4.33–9.92)	**<0.001**
Yes	252 (21.8)	92 (56.1)				
Age						
Mean	48.6	54.4	1.03 (1.01–1.04)	**<0.001**	1.03 (1.02–1.04)	**<0.001**
Median	49.0	58.0				
SD	15.6	13.8				
Min	18	22				
Max	75	75				
Female						
No	234 (20.3)	27 (16.5)	1.29 (0.83–2.00)	0.255	0.81 (0.51–1.29)	0.373
Yes	921 (79.7)	137 (83.5)				
Smoking history						
Never	714 (61.8)	88 (53.7)	1.40 (1.01–1.94)	**0.046**	0.74 (0.52–1.05)	0.088
Yes, at some point	441 (38.2)	76 (46.3)				

*Notes*: Each study individual involves in one of the sections. Section 4 members were selected first. Bold values indicate *p* value of 0.05 or less was considered statistically significant.

Abbreviations: AS, asthma; AR, allergic rhinitis; AC, allergic conjunctivitis; AD, atopic dermatitis; FA, food allergy; SD, standard deviation.
