# Supplementary material for: Allergic multimorbidity is associated with self‐reported anaphylaxis in adults—A cross‐sectional questionnaire study
Source: Clin Transl Allergy. 2022 Jul 21;12(7):e12184. doi: 10.1002/clt2.12184 (PMC9301681; doi:10.1002/clt2.12184)
Supplement: Supplementary file 1 — Supporting Information S1 [file CLT2-12-e12184-s001.docx]

**Supplement**

Table S1. The severity of disease by study individuals with self-reported anaphylaxis with FA/AR/AC/AD and with/without AS.

| **In the past 12 months** | N = 164 |
| --- | --- |
| **Oral corticosteroids**  occasionally *n (%) number of periods [min-max]*  regularly *n (%)* | 57 (35) [1-10]  6 (4) |
| **Antihistamines**  occasionally *n (%)*  regularly *n (%)* | 65 (40)  85 (52) |
| **Epinephrine injection**  once *n (%)*  at least twice *n (%)* | 8 (5)  3 (2) |
| **Emergency room visits**  allergies *n (%) [min-max]*  asthma *n (%) [min-max]* | 20 (12) [1-3]  24 (15) [1-12] |

Table S2. The anaphylaxis risk of allergic multimorbidity subgroups exhibited in the study population.

|  |  |  | **Univariate** |  |
| --- | --- | --- | --- | --- |
| **Allergic multimorbidity subgroups** | **No anaphylaxis (n = 1155)**  **with the allergic multimorbidity subgroup**  **n (%)** | **Anaphylaxis (n = 164)**  **with the allergic multimorbidity subgroup**  **n (%)** | **OR (CI 95 %)** | **P value** |
| AS | 0 (0) | 0 (0) | - | - |
| AR | 140 (12.1) | 4 (2.4) | 0.18 (0.07–0.50) | **0.001** |
| AS+AR | 301 (26.1) | 19 (11.6) | 0.37 (0.23–0.61) | **< 0.001** |
| AC | 8 (0.7) | 0 (0) | - | - |
| AS+AC | 8 (0.7) | 0 (0) | - | - |
| AR+AC | 49 (4.2) | 4 (2.4) | 0.56 (0.20–1.59) | 0.277 |
| AS+AR+AC | 74 (6.4) | 7 (4.3) | 0.65 (0.30–1.44) | 0.289 |
| AD | 21 (1.8) | 2 (1.2) | 0.67 (0.16–2.87) | 0.586 |
| AS+AD | 40 (3.5) | 4 (2.4) | 0.70 (0.25–1.97) | 0.497 |
| AR+AD | 36 (3.1) | 1 (0.6) | 0.19 (0.03–1.40) | 0.103 |
| AS+AR+AD | 72 (6.2) | 8 (4.9) | 0.77 (0.37–1.63) | 0.497 |
| AC+AD | 2 (0.2) | 1 (0.6) | 3.54 (0.32–39.22) | 0.303 |
| AS+AC+AD | 3 (0.3) | 1 (0.6) | 2.36 (0.24–22.78) | 0.459 |
| AR+AC+AD | 24 (2.1) | 0 (0) | - | - |
| **AS+AR+AC+AD** | 36 (3.1) | 11 (6.7) | **2.24 (1.11–4.48)** | **0.024** |
| FA | 3 (0.3) | 1 (0.6) | 2.36 (0.24–22.78) | 0.459 |
| **AS+FA** | 38 (3.3) | 11 (6.7) | **2.11 (1.06–4.22)** | **0.034** |
| AR+FA | 18 (1.6) | 1 (0.6) | 0.39 (0.05–2.92) | 0.358 |
| **AS+AR+FA** | 63 (5.5) | 16 (9.8) | **1.87 (1.06–3.33)** | **0.032** |
| AC+FA | 2 (0.2) | 0 (0) | - | - |
| AS+AC+FA | 6 (0.5) | 1 (0.6) | 1.18 (0.14–9.82) | 0.882 |
| AR+AC+FA | 18 (1.6) | 2 (1.2) | 0.78 (0.18–3.39) | 0.740 |
| **AS+AR+AC+FA** | 30 (2.6) | 10 (6.1) | **2.44 (1.17–5.08)** | **0.018** |
| AD+FA | 8 (0.7) | 1 (0.6) | 0.88 (0.11–7.08) | 0.904 |
| AS+AD+FA | 18 (1.6) | 6 (3.7) | 2.40 (0.94–6.13) | 0.068 |
| AR+AD+FA | 17 (1.5) | 1 (0.6) | 0.41 (0.05–3.11) | 0.389 |
| **AS+AR+AD+FA** | 41 (3.5) | 18 (11.0) | **3.35 (1.88–5.99)** | **< 0.001** |
| AC+AD+FA | 1 (0.1) | 1 (0.6) | 7.08 (0.44–13.74) | 0.167 |
| AS+AC+AD+FA | 1 (0.1) | 0 (0) | - | - |
| AR+AC+AD+FA | 25 (2.2) | 4 (2.4) | 1.13 (0.39–3.29) | 0.823 |
| **AS+AR+AC+AD+FA** | 52 (4.5) | 29 (17.7) | **4.56 (2.80–7.42)** | **< 0.001** |

AS = asthma, AR = allergic rhinitis, AC = allergic conjunctivitis, AD = atopic dermatitis, FA = food allergy

OR compares “Yes” to “No” (reference).
